# Is the measurement of delivered dose a good performance measure? A characterization of delivered and respirable delivered dose in two brands of jet nebulizer

**DOI:** 10.1186/2045-7022-3-S1-P10

**Published:** 2013-05-03

**Authors:** Mariam Rehman, Adam Metcalf, Ross Hatley

**Affiliations:** 1 Respironics Respiratory Drug Delivery (UK) Ltd, Philips Home Healthcare Solutions, UK

## Background

Use of a nebulizer facilitates rapid administration of high doses of drug, which is desirable for patients presenting with acute severe asthma. Breath-enhanced jet nebulizers direct the patient**’**s air flow through the nebulizer handset during inhalation producing aerosol output with a more favorable respirable profile. SideStream Plus (SS+; Philips Respironics) and Nebutech (NB; Salter Labs) are breath-enhanced jet nebulizers, designed to produce a high delivered dose in a short treatment time. A previous *in vitro* characterization of delivered dose and droplet size distribution of arformoterol nebulized via the SS+ and the NB indicated that the SS+ performed favorably, compared with the NB.[1] We present results of an *in vitro* characterization of salbutamol sulphate (albuterol sulfate) delivery via the SS+ nebulizer and the NB nebulizer.

## Method

Each nebulizer was filled with 3 ml salbutamol sulphate (5 mg/2.5 ml) and run with a driving flow of 8 l/min into a CEN adult tidal breathing pattern (Vt; 500 ml, 15 BPM, I:E ratio; 1:1). The nebulizers were run in triplicate to sputter point and sputter point plus 60 s. Dose delivered to a filter (delivered dose; DD), placed between the nebulizer and breathing emulator, was quantified using high performance liquid chromatography and expressed as a µl solution equivalent. A laser diffractor (Malvern Spraytec) was used to assess fine particle fraction (FPF) and mass median diameter (MMD) after 60 s nebulization time. Fine particle dose (FPD) was calculated (FPF x delivered dose).

## Results

The results are shown in Table 1

**Table 1:** 

Experimental parameter	SS+	NB
MMD (µm)	3.2	5.6
FPF (%<5 µm)	74.2	43.7
DD; Sputter (µl solution)	432	424
FPD; Sputter (µl solution)	321	185
DD; Sputter + 60 s (µl solution)	542	594
FPD; Sputter + 60 s (µl solution)	402	260

## Conclusion

The total DD from the 2 nebulizers was comparable. Aerosol delivered via the SS+ nebulizer had a lower MMD and higher FPF, compared with that delivered via the NB nebulizer. This resulted in a 74% higher FPD delivered to sputter, and a 55% higher FPD delivered to sputter plus 60 s from the SS+ nebulizer compared with the NB nebulizer. The FPD represents respirable aerosol, and is therefore more clinically relevant than the DD. These results are consistent with previous work, and indicate that a high respirable dose of aerosol is produced by the SS+ nebulizer [1].
